# The Role of Physiological Vitamin C Concentrations on Key Functions of Neutrophils Isolated from Healthy Individuals

**DOI:** 10.3390/nu11061363

**Published:** 2019-06-17

**Authors:** Stephanie M. Bozonet, Anitra C. Carr

**Affiliations:** Department of Pathology and Biomedical Science, University of Otago, Christchurch, P.O. Box 4345, Christchurch 8140, New Zealand; stephanie.bozonet@otago.ac.nz

**Keywords:** vitamin C, ascorbate, immunity, neutrophils, chemotaxis, neutrophil extracellular traps

## Abstract

Vitamin C (ascorbate) is important for neutrophil function and immune health. Studies showing improved immune function have primarily used cells from scorbutic animals or from individuals with infectious conditions or immune cell disorders. Few studies have focused on the requirements of neutrophils from healthy adults. Therefore, we have investigated the role of vitamin C, at concentrations equivalent to those obtained in plasma from oral intakes (i.e., 50–200 µmol/L), on key functions of neutrophils isolated from healthy individuals. Cells were either pre-loaded with dehydroascorbic acid, which is rapidly reduced intracellularly to ascorbate, or the cells were activated in the presence of extracellular ascorbate. We measured the effects of enhanced ascorbate uptake on the essential functions of chemotaxis, oxidant production, programmed cell death and neutrophil extracellular trap (NET) formation. We found that neutrophils isolated from healthy individuals already had replete ascorbate status (0.35 nmol/10^6^ cells), therefore they did not uptake additional ascorbate. However, they readily took up dehydroascorbic acid, thus significantly increasing their intracellular ascorbate concentrations, although this was found to have no additional effect on superoxide production or chemotaxis. Interestingly, extracellular ascorbate appeared to enhance directional mobilityin the presence of the chemoattractant formyl-methionyl-leucyl-phenylalanine (fMLP). Stimulation of the cells in the presence of ascorbate significantly increased intracellular ascorbate concentrations and, although this exhibited a non-significant increase in phosphatidylserine exposure, NET formation was significantly attenuated. Our findings demonstrate the ability of neutrophils to regulate their uptake of ascorbate from the plasma of healthy humans to maintain an optimal level within the cell for proper functioning. Higher oral intakes, however, may help reduce tissue damage and inflammatory pathologies associated with NET formation.

## 1. Introduction

Vitamin C (ascorbate) is an essential nutrient which humans must acquire daily through the diet. It is a potent antioxidant, able to protect important biomolecules from the damaging effects of oxidants produced endogenously, during metabolism and inflammation, and from the environment [1]. It is also an essential cofactor for many enzymes involved in important biosynthetic and regulatory processes, including collagen and carnitine biosynthesis [2], hormone production [3], gene transcription [4] and epigenetic regulation [5]. Severe, prolonged vitamin C deficiency results in scurvy, a potentially fatal disease characterized by the breakdown of collagenous tissue. This leads to impaired wound healing and compromised immunity, leaving the individual vulnerable to life-threatening infections [6]. Severe respiratory infections, such as pneumonia, are a common complication of severe vitamin C deficiency and are one of the most common causes of mortality in vitamin C-deficient individuals [7]. Additionally, the enhanced oxidative stress and inflammation observed during infections can result in significant depletion of vitamin C due to increased requirements for the vitamin, and this depletion is also apparent in vital immune cells such as neutrophils [8].

In contrast to plasma ascorbate levels, which reflect what has been absorbed from the diet, neutrophils concentrate the vitamin through active uptake via the sodium-dependent vitamin C transporter-2 (SVCT2). Intracellular concentrations of ascorbate can therefore be in the millimolar range and are thought to be indicative of enhanced requirement by that particular cell type [9]. Once activated, neutrophils can further increase their intracellular concentration by the uptake of oxidized ascorbate, dehydroascorbate (DHA), via glucose transporters (GLUTs). Once inside the cell, DHA is immediately reduced to ascorbate, maintaining the concentration gradient and allowing further influx of DHA, thus resulting in increases of intracellular ascorbate by up to 20-fold [9]. This dramatic accumulation of ascorbate by activated neutrophils is thought to indicate an important requirement for their function.

Neutrophils are important mediators of the innate immune response whose primary function is to destroy invading pathogens. Upon detection of microbial products and inflammatory cytokines these cells migrate to sites of infection, a process known as chemotaxis, whereupon they engulf the pathogenic organisms, sequestering them in vesicles where they can be safely destroyed [10]. Microbial killing occurs by enzymatic as well as oxidative mechanisms, and it is thought that the antioxidant function of ascorbate is important in protecting the neutrophil during this process [11]. Attenuated chemotaxis, phagocytosis, oxidant production and microbial killing have been observed in neutrophils isolated from scorbutic animals, compared with controls, and dysfunctional activities were reversed by supplementation with ascorbate [12,13,14].

Following activation, neutrophils undergo a form of programmed cell death, known as phagocytosis-induced cell death, whereby oxidants generated in the phagosome result in phosphatidylserine exposure which facilitates clearance of the spent cells by macrophages [15]. This process occurs within hours of neutrophil stimulation and is critical for effective resolution of the immune response. Impaired resolution of the inflammatory response can result in tissue damage due to the release of proteases and pro-inflammatory mediators from necrotic cells, and is associated with autoimmune conditions and multiple organ failure [16,17]. Neutrophils can also generate extracellular traps (NETs), whereby DNA strands dotted with histones, proteases and oxidases are ejected from the cell [18]. Although NETs can trap invading pathogens, they are nonspecific and potentially inflammatory, damaging host tissues and resulting in various pathologies [19,20]. Dysfunctional neutrophil cell death and diminished uptake by macrophages has been reported in cells isolated from ascorbate-deficient gulonolactone oxidase (Gulo) knockout mice [21]. Furthermore, it has been demonstrated that vitamin C can decrease NET formation in septic Gulo knockout mice and in stimulated human neutrophils [22].

Although a number of studies have been carried out to explore the effects of vitamin C supplementation on neutrophil function, these have often used individuals with infectious or allergic conditions, or abnormal neutrophil function e.g., those with chronic granulomatous disease or Chédiak–Higashi syndrome [6]. However, very little information exists with respect to the potential for vitamin C supplementation, which provides physiological plasma concentrations, to enhance the function of neutrophils in healthy individuals [23]. Evidence suggests that plasma levels of ascorbate should be maintained above 50 μmol/L, a threshold viewed as adequate to maintain health [24]. In healthy individuals, a daily intake of 100–200 mg provides adequate to saturating steady-state levels of ascorbate in the plasma (i.e., 50–80 μmol/L) [25,26]. An intake of 200 mg/day is a suggested dietary target to reduce the risk of chronic disease [27], and meta-analyses have indicated that a vitamin C intake of at least 200 mg/day can decrease the risk of acquiring respiratory infections [28,29]. However, gram doses of vitamin C are required once an infection has taken hold, due to increased requirements for the vitamin [28,29,30]. Gram doses of oral vitamin C have been shown to provide peak plasma ascorbate concentrations of greater than 150 μmol/L [31].

The aim of this study was, therefore, to investigate whether increasing the intracellular ascorbate concentration, using physiologically relevant concentrations of ascorbate (i.e., 50–200 μmol/L), could improve essential functions of neutrophils isolated from healthy volunteers. The participants in this study were not asked to modify their eating habits in any way, nor were they fasting when blood was drawn, to more closely resemble normal day-to-day conditions. Neutrophils were isolated from whole blood and the intracellular ascorbate concentrations were measured before and after incubation with ascorbate or DHA. Analyses were carried out to determine the effects of pre-loading neutrophils with ascorbate, as well as co-incubation in the presence of extracellular ascorbate during activation, on the primary functions of chemotaxis, oxidant generation, programmed cell death and NET formation.

## 2. Materials and Methods

### 2.1. Materials

Vacutainer tubes were from Becton Dickinson (Auckland, NZ) and heparin was acquired through the Christchurch Hospital Pharmacy (Christchurch, NZ). Cell culture reagents (RPMI 1640 medium without phenol red, foetal bovine serum (FBS), penicillin and streptomycin), Sytox Green™ and the Annexin V-FITC antibody were from Life Technologies (Auckland, NZ). Ficoll-Hypaque was from Global Sciences (Auckland, NZ) and Calcein AM was from In Vitro Technologies (Auckland, NZ). Fluoroblok™ microtitre plates (96-well, 0.3 μm pore size) were from Becton Dickinson (Auckland, NZ). All other reagents (dextran, glucose, perchloric acid (PCA), dithiothreitol (DTT), diethylenetriaminepentaacetic acid (DTPA) cytochrome *c*, phorbol 12-myristate 13-acetate (PMA), catalase and formyl-methionyl-leucyl-phenylalanine (fMPL) were from Merck (formerly Sigma, Auckland, NZ).

### 2.2. Neutrophil Isolation

The collection of blood samples was approved by the Southern Health and Disability Ethics Committee, New Zealand (URA/06/12/083/AM05) and informed consent was obtained from all blood donors. Blood from healthy donors was collected into heparin tubes and neutrophils were isolated as described previously [32]. Briefly, neutrophils were obtained using Ficoll–Hypaque and dextran sedimentation, followed by hypotonic erythrocyte lysis. Neutrophils were resuspended in buffer (Hanks Buffered Saline Solution, HBSS, or phosphate buffered saline, PBS, with 5 mM glucose) or medium (RPMI with 2% or 10% fetal bovine serum) as indicated.

### 2.3. Ascorbate and DHA Loading of Neutrophils

Reagent ascorbate and DHA were prepared in HBSS for cell uptake experiments. The concentration of ascorbate was calculated from spectrophotometric measurement at 265 nm (ε = 14,500 M^−1^ cm^−1^). The DHA was first reduced to ascorbate by incubation for 5 min at room temperature with DTT (2.5 mM). Due to the instability of DHA [33], this was made freshly before each use, was kept on ice at all stages, and was added immediately to the cells following standardization.

Cells were dispensed into sterile 1.7 mL tubes at 5 × 10^6^/mL with either ascorbate or DHA (at indicated concentrations of 50–200 μmol/L, in 1 mL total volume) for indicated times (i.e., 7–90 min). Tubes were rotated gently end-over-end (6 rmp) at 37 °C. After incubation, the cells were centrifuged at 2300 rpm for 5 min and washed twice in PBS to remove any extracellular ascorbate or DHA before HPLC analysis.

### 2.4. Ascorbate Uptake by Stimulated Neutrophils

Neutrophils were resuspended at 1 × 10^6^/mL in RPMI 1640 medium containing 10% FBS (2 mL final volume) and incubated in 12-well culture dishes, at 37 °C in a 5% CO_2_ atmosphere. The cells were incubated in the presence of extracellular ascorbate (200 µmol/L), with and without PMA (100 ng/mL) for the indicated times (i.e., 10–60 min), and intracellular ascorbate was measured. Cells were similarly stimulated for 45 min in the presence of increasing concentrations of extracellular ascorbate (i.e., 50–200 µmol/L), in the presence of PMA (100 ng/mL), and intracellular ascorbate was measured.

### 2.5. Intracellular Ascorbate Analysis by HPLC

For intracellular ascorbate measurement, neutrophils (2–5 × 10^6^) were pelleted by centrifugation at 10,000 rpm for 1 min at room temperature. Cells were resuspended in PBS (100 μL) and an equal volume of ice cold PCA (0.54 M, containing the metal ion chelator DTPA, 100 μmol/L) was added. This was vortexed and samples kept on ice for 10 min before centrifugation (12,000 rpm for 2 min at 4 °C) to remove protein. Supernatants were stored at −80 °C and analyzed by HPLC with electrochemical detection as described previously [34].

### 2.6. Chemotaxis

Neutrophil chemotaxis was measured using a method modified from Kuijpers et al. [35]. Cells were resuspended at 5 × 10^6^/mL in PBS with glucose (5 mM, PBS + G) and labelled by incubation with Calcein AM (1 μM) for 30 min at 37 °C. After washing twice with PBS + G, cells were resuspended in PBS + G at 1 × 10^6^/mL, and 50 μL (50,000 cells) was delivered to the top compartment of a 96-well Fluoroblok™ microtitre plate. PBS + G with or without chemoattractant fMLP (100 nM), and with or without ascorbate, was added to the bottom compartment (225 μL/well). Directed cell migration into the lower chamber was monitored by measuring fluorescence (excitation 485 nm, emission 535 nm) over a 30 min period at 37 °C. The level of chemotaxis was determined as the amount of fluorescence measured in the lower compartment of the chamber after 30 min incubation.

### 2.7. Superoxide Generation

As described previously [36], the rate of superoxide generation by activated neutrophils was measured indirectly as a function of cytochrome *c* reduction. Neutrophils (0.5 × 10^6^) were stimulated with PMA (100 ng/mL) in the presence of catalase (20 μg/mL) and cytochrome *c* (40 μM). Activity (μmol superoxide/min/10^6^ cells) was calculated from the change in absorbance at 550 nm (over 5 min at 37 °C) using the extinction coefficient, 21.1 × 10^3^ M^−1^ cm^−1^.

### 2.8. Phosphatidylserine Exposure

The effect of ascorbate uptake on cell surface phosphatidylserine exposure of stimulated neutrophils was assessed using Annexin V-FITC binding and flow cytometry (Cytomics FC 500 Flow Cytometry system, Beckman Coulter Inc., North Shore City, New Zealand). After incubation in RPMI containing 10% FBS, with extracellular ascorbate (0, 100 or 200 µmol/L), in the presence or absence of PMA (100 ng/mL), cells (1 × 10^6^ cells/mL) were resuspended in buffer containing Annexin V-FITC according to the manufacturer’s instructions and the fluorescence of 10,000 cells was analyzed.

### 2.9. NET Production

NET production by PMA-stimulated neutrophils was measured using a fluorescent plate assay method [37]. Neutrophils (1 × 10^6^/mL) were resuspended in RPMI 1640 medium, containing 2% FBS, and 100 µL/well was added, in triplicate, to a black 96-well tissue culture plate. Cells were incubated with extracellular ascorbate (0, 100 or 200 µmol/L) in the presence or absence of PMA (20 nM) for 4 h at 37 °C with 5% CO_2_. Extracellular DNA (indicative of NET production) was then stained with Sytox Green™ (5 µmol/L) and the fluorescence measured (excitation 485 nm, emission 520 nm) using a PolarStar™ plate reader (BMG LABTECH, Alphatech Systems Ltd. Auckland, NZ). NET production was calculated as the difference in fluorescence between stimulated and unstimulated cells at 4 h.

### 2.10. Statistical Analysis

Data are presented as mean ± standard error of the mean (SEM), and analysis of paired data was carried out using two-tailed Student’s t-test with significance determined as (*) *p* < 0.05 (SigmaPlot, Systat Software Inc., San Jose, CA, USA).

## 3. Results

### 3.1. Ascorbate and DHA Loading of Neutrophils

**Figure 1:**
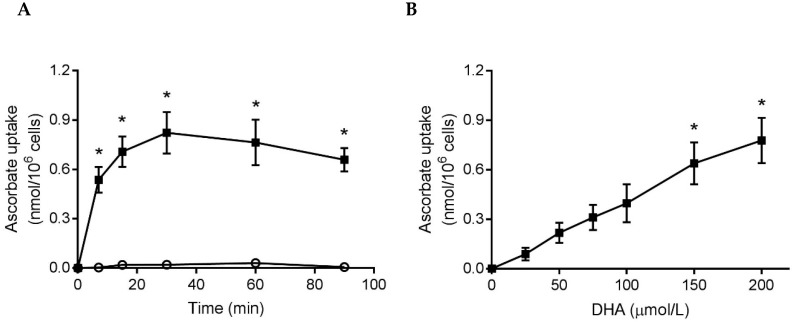
Uptake of ascorbate by neutrophils isolated from healthy individuals. (**A**) Cells were incubated with ascorbate (○) or DHA (■), 200 µmol/L, in HBSS for up to 90 min. Intracellular ascorbate was measured by HPLC and expressed as the increase above that of cells processed at 0 min (0.38 ± 0.05 nmol/10^6^ cells), *n* = 3. (**B**) Cells were incubated with increasing concentrations of DHA for 15 min and the intracellular ascorbate concentration expressed as the increase above that of cells in HBSS (0.32 ± 0.01 nmol/10^6^ cells), *n* = 3. Data represent mean ± SEM, * *p* < 0.05.

To ascertain whether neutrophils from healthy individuals would uptake additional ascorbate, we incubated cells with either ascorbate or DHA (200 µmol/L), for up to 90 min, and measured the intracellular ascorbate concentration. Mean plasma ascorbate concentrations were 82 ± 5 µmol/L (*n* = 7), indicating saturation, and the mean baseline concentration of the neutrophils was 0.35 ± 0.01 nmol/10^6^ cells (*n* = 13). The isolated neutrophils did not uptake ascorbate, but did uptake DHA which increased their mean intracellular ascorbate concentration by 0.82 ± 0.13 nmol/10^6^ cells compared to cells not incubated with DHA (Figure 1A). Incubation of cells for 15 min in the presence of increasing concentrations of DHA (25–200 µmol/L) showed a dose-dependent uptake (Figure 1B).

### 3.2. Chemotaxis

**Figure 2:**
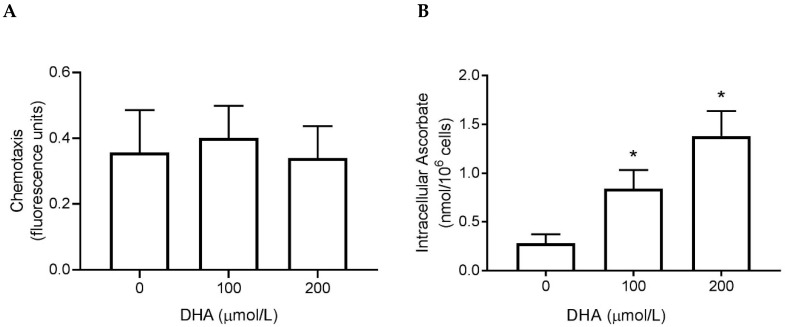
The effect of ascorbate uptake on neutrophil chemotaxis. (**A**) The directional motility of preloaded cells towards a microbial chemoattractant (fMLP). Graphical representation of the fluorescence detected in the lower assay compartment after 30 min. (**B**) The intracellular ascorbate concentration of the preloaded cells was measured using HPLC. Data represent mean ± SEM, *n* = 3, * *p* < 0.05.

We measured the chemotactic response of neutrophils to a microbial signal (fMLP) after pre-loading the cells with DHA to increase their intracellular ascorbate concentration. There was no effect on chemotaxis (Figure 2A), despite increasing the intracellular ascorbate concentration (Figure 2B).

**Figure 3:**
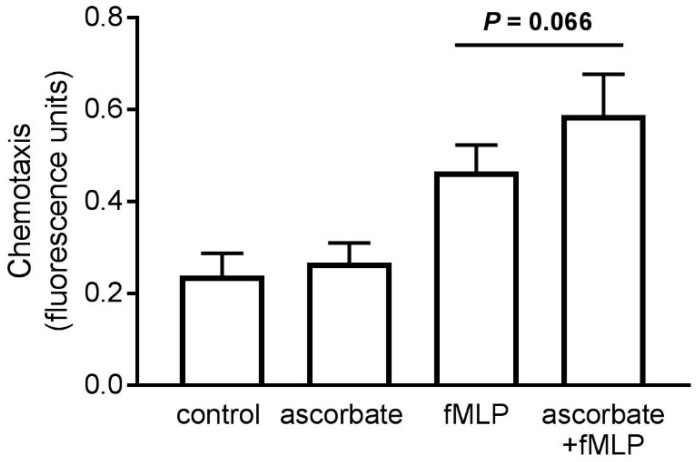
The effect of extracellular ascorbate on neutrophil chemotaxis. The directional motility of cells towards fMLP alone, or with extracellular ascorbate (100 µmol/L), was measured. Graphical representation of the fluorescence detected in the lower assay compartment after 30 min. Data represent mean ± SEM, *n* = 3.

We also measured the effect of extracellular ascorbate on the directional motility of neutrophils. We found that adding ascorbate (100 µmol/L) to the lower compartment further enhanced fMLP-stimulated directional motility of the cells, although this did not quite reach statistical significance (Figure 3).

### 3.3. Superoxide Generation

**Figure 4:**
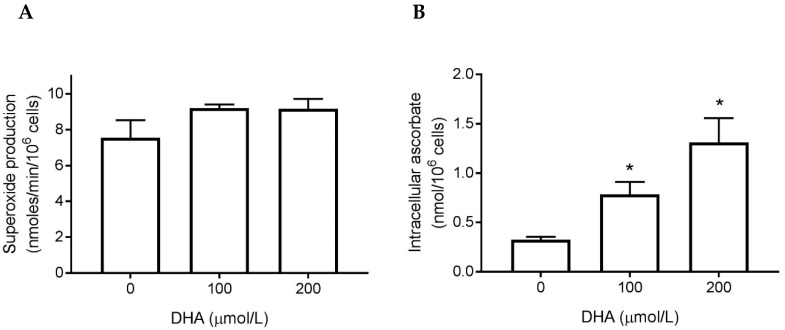
The effect of ascorbate uptake on superoxide generation by stimulated neutrophils. (**A**) Cells pre-loaded with DHA were stimulated with PMA (100 ng/mL) and the rate of superoxide generation measured over 5 min. (**B**) The intracellular ascorbate concentration of preloaded cells was measured by HPLC. Data represent mean ± SEM, *n* = 3. * *p* < 0.05.

After pre-loading cells with DHA, superoxide generation was assessed as a measure of the oxidative burst, and potential microbicidal activity, of the isolated neutrophils. The cells were stimulated with the phorbol ester PMA, an agonist of the protein kinase C signal transduction pathway that results in superoxide generation by the NADPH oxidase complex. We observed a small, non-significant increase in oxidant production (Figure 4A) with increasing intracellular ascorbate concentration (Figure 4B). 

### 3.4. Ascorbate Uptake by Stimulated Neutrophils

**Figure 5:**
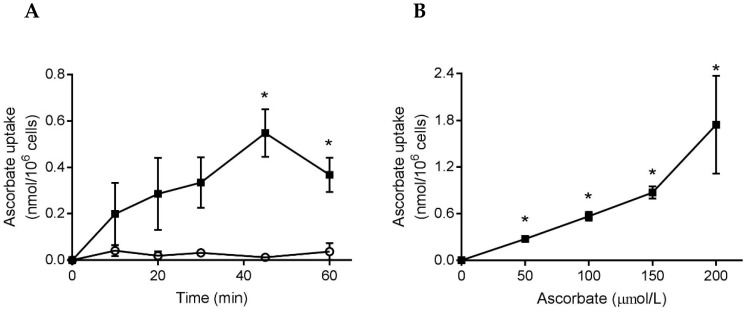
Ascorbate uptake by stimulated neutrophils. (**A**) Cells were incubated with extracellular ascorbate (100 µmol/L) for the indicated times, in the absence (○) or presence (■) of PMA (100 ng/mL). The time zero ascorbate concentration was 0.34 ± 0.134 nmol/10^6^ cells, *n* = 4. (**B**) Cells were incubated with increasing concentrations of ascorbate for 45 min in the presence of PMA (100 ng/mL). The starting ascorbate concentration was 0.29 ± 0.11 nmol/10^6^ cells, *n* = 3. The intracellular ascorbate concentration was measured by HPLC. Data represent mean ± SEM, * *p* < 0.05.

We assessed the ability of isolated neutrophils to uptake ascorbate, provided in the medium at a physiologically relevant concentration (100 μmol/L), over time. As expected, there was no increase in intracellular ascorbate without stimulation, but a mean increase of 0.55 ± 0.08 nmol/10^6^ cells at 45 min was observed in the presence of PMA (Figure 5A). In support of this, ascorbate uptake by stimulated cells increased in a dose-dependent manner as the extracellular ascorbate concentration increased (Figure 5B). The mean intracellular ascorbate concentration, at 200 μmol/L, increased by 1.74 ± 0.51 nmol/10^6^ cells after 45 min.

### 3.5. Effect of Ascorbate on Phosphatidylserine Exposure

**Figure 6:**
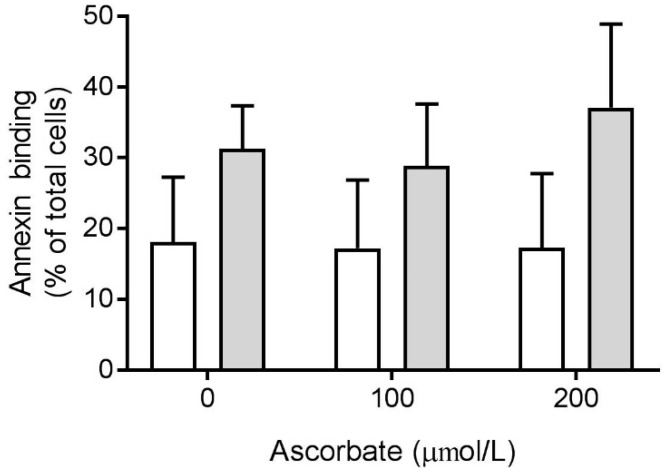
Effect of ascorbate on phosphatidylserine exposure of activated neutrophils. Cells were incubated with extracellular ascorbate (0, 100 or 200 µmol/L) and stimulated with PMA (100 ng/mL) for 2 h (white bars) or 4 h (grey bars). Phosphatidylserine exposure was measured by flow cytometry with Annexin-V FITC. Data represent mean ± SEM, *n* = 5.

Having established that the isolated neutrophils significantly increased their ascorbate content when stimulated in the presence of extracellular ascorbate (Figure 5), we investigated the effect of this on phosphatidylserine exposure as a measure of cell death and clearance. Cells were stimulated with PMA (100 ng/mL) for 2 or 4 h, in the presence of ascorbate, and the amount of Annexin-V FITC binding to externalized phosphatidylserine was measured by flow cytometry. There was no statistically significant effect of increased intracellular ascorbate on phosphatidylserine exposure after 2 or 4 h (Figure 6).

### 3.6. Effect of Ascorbate on NET Production

**Figure 7:**
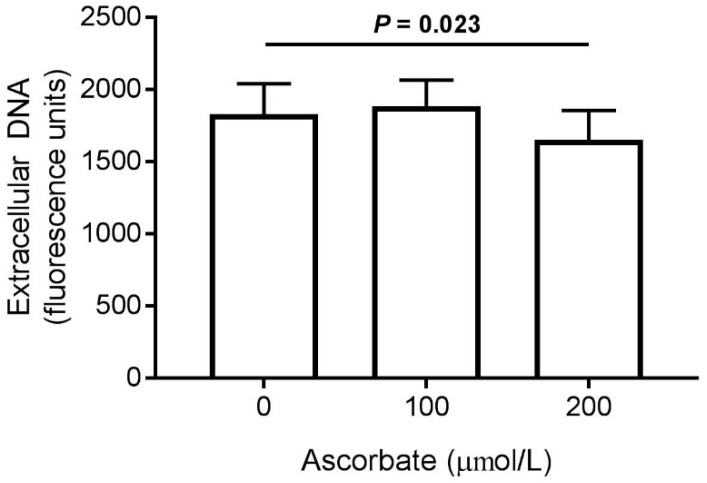
Effect of ascorbate on NET production by activated neutrophils. Cells were incubated with extracellular ascorbate (0, 100 or 200 µmol/L) and stimulated with PMA (2 μg/mL) for 4 h. Extracellular DNA was stained with SytoxGreen™ and fluorescence measured as a marker of NET production. Data represent mean ± SEM, *n* = 5.

We assessed the amount of DNA released by stimulated neutrophils, incubated in the presence of ascorbate, as an indicator of NET production. There was an attenuating effect of ascorbate on extracellular DNA released by neutrophils at the highest dose (200 μmol/L), which was statistically significant (Figure 7).

## 4. Discussion

A number of studies have investigated the effects of vitamin C supplementation on neutrophils in conditions of vitamin C depletion, such as infectious disease, but little information exists with regard to neutrophils isolated from healthy adults with adequate plasma ascorbate levels [6]. In earlier trials we showed that supplementing participants who had a low dietary vitamin C intake could increase their neutrophil ascorbate concentrations [23,38,39], and a positive effect on neutrophil chemotaxis and oxidant production was observed [23]. In the current study we found that the baseline neutrophil ascorbate concentrations were comparable to those measured after supplementing participants with saturating levels of vitamin C [23]. Thus, the cells were fully replete, as confirmed by the saturating plasma concentrations, and would only take up additional ascorbate in the form of DHA when unstimulated. This is not unexpected given that ascorbate import by SVCT2 is regulated according to the intracellular concentration, whereas DHA enters the cells via GLUTs and is subsequently converted to ascorbate, thus maintaining a concentration gradient which facilitates further DHA uptake [9]. It is therefore possible to artificially raise intracellular ascorbate levels in already replete neutrophils. Although this is unlikely to occur under normal physiological conditions, as DHA is present at negligible levels in plasma [33,40], the oxidizing microenvironment of inflammatory loci could potentially result in extracellular ascorbate being oxidized to DHA in close proximity to sequestered neutrophils.

In our previous intervention study [23] we observed an increase in chemotaxis and superoxide production following supplementation of participants with low vitamin C status, yet there were no effects of ascorbate pre-loading on these functions in the current in vitro study. This may be due to baseline ascorbate levels in the isolated cells being already adequate for effective chemotaxis and oxidant production and suggests that maintaining neutrophil ascorbate levels around 0.35 nmol/10^6^ cells may be an important factor in maintaining a healthy immune response. Another in vitro study reported ascorbate and DHA-dependent enhancement of superoxide generation by cells isolated from healthy non-fasting volunteers, however, they did not measure baseline cell ascorbate concentrations [41]. Anderson and Lukey [42] showed that ascorbate exhibited a dual role in isolated phagocytes, by enhancing intracellular oxidant generation, whilst scavenging oxidants released extracellularly, thus potentially protecting host tissues against oxidative damage.

Interestingly, although we saw no effect of ascorbate pre-loading on chemotaxis, we did observe a trend towards enhanced directional motility of the cells in response to extracellular ascorbate in the presence of the chemoattractant fMLP. This finding is comparable to an earlier study, which indicated that ascorbate, in combination with interleukin-8, enhanced the directional motility of neutrophils [43]. The mechanisms involved in ascorbate-stimulated chemotaxis are not yet understood, although enhanced microtubule formation has been suggested [44]. Recently, it was proposed that neutrophil motility could be used as an indication of vitamin C intake requirements [45], however, more research would need to be carried out in neutrophils that are ascorbate deficient in order to determine the minimum amounts of vitamin C required for optimal chemotactic activity.

In contrast to resting neutrophils, we showed that cells stimulated with PMA took up ascorbate from the medium in a time- and concentration-dependent manner. However, this provided only a non-significant increase in phosphatidylserine exposure in PMA-stimulated cells. In our earlier intervention study, there was no effect of vitamin C supplementation on spontaneous (unstimulated) neutrophil apoptosis, and it was concluded that the baseline concentrations of ascorbate may have already been sufficient to support programmed cell death [23]. It should be noted that neutrophils saturate at much lower vitamin C doses (~100 mg/day) than plasma saturation (~200 mg/ day) [25]. In the current study, phosphatidylserine exposure after stimulation of cells in the presence of physiological concentrations of ascorbate was minimal, which suggests that the level of intracellular ascorbate upon isolation of the cells was adequate to allow efficient phosphatidylserine exposure and clearance of stimulated cells. Sharma et al. [41] observed a significant increase in neutrophil phosphatidylserine exposure following incubation with DHA and a bacterial stimulant, however, baseline ascorbate concentrations were not determined in that study. Efficient clearance of spent neutrophils is critical for maintaining physiological homeostasis and preventing tissue damage associated with cell necrosis and poor uptake by macrophages [16,46].

One function where we did see a significant effect of increased ascorbate uptake by stimulated cells was in NET production; when cells were co-incubated with ascorbate and PMA, NET formation was significantly decreased. NET components such as histones can act as damage-associated molecular pattern proteins (DAMP) which stimulate inflammation and have been linked with autoimmune conditions and organ damage in sepsis [19,20]. Indeed, sepsis patients have elevated levels of circulating cell-free DNA [47,48] and a preclinical study has shown ascorbate to be a negative regulator of NET formation in a murine sepsis model and in human neutrophils in vitro [22]. Interestingly, the transcription factor hypoxia-inducible factor (HIF)-1 has been associated with NET production [49] and ascorbate is a key cofactor for the enzymes that downregulate HIF-1 [50]. HIF-1 has also been shown to prolong the survival of neutrophils in regions of localized hypoxia [51], thus, the regulation of HIF-1 may be one mechanism by which ascorbate affects neutrophil function and death [21].

For our earlier intervention study, the participants were screened and selected for having low plasma ascorbate and also underwent a 3-week lead-in period, during which time they limited their vitamin C intake still further [23]. Despite this, neutrophil ascorbate levels remained at around 0.21 nmol/10^6^ cells, even when plasma levels fell well below the adequate threshold to 26 μmol/L. This supports the premise that neutrophils retain their ascorbate levels, even when plasma levels drop, because it serves critical roles within the cell. During infectious episodes, however, neutrophils are known to become depleted of ascorbate, and this appears to impact negatively on their functions [6,8]. Supplementation of individuals with respiratory infections with saturating doses of vitamin C results in enhanced neutrophil ascorbate status and improved neutrophil functions in many cases [6,8,52].

Overall, our study has shown that although a low dose vitamin C intake, which provides <100 µmol/L plasma concentration, may not impact on neutrophil functions in healthy individuals, higher (gram) doses may provide sufficiently high peak concentrations to impact on some functions, such as NET formation, which was decreased in the presence of 200 µmol/L ascorbate. Two intervention studies have demonstrated beneficial effects of gram dose vitamin C supplementation on the function of neutrophils from healthy donors. In a double-blind cross-over trial, enhanced chemotaxis was observed following supplementation with 2 g/day for one week [53]. In a similar study, chemotaxis was enhanced in neutrophils isolated from healthy adults after ingestion of 2 or 3 g/day of vitamin C for one week [54]. Interestingly, 1 g/day had no effect when administered orally, however the same group observed increased neutrophil motility 1 h after a single 1 g intravenous dose of ascorbate, which is known to provide significantly higher peak ascorbate concentrations [31,55].

## 5. Conclusions

In this study we have shown that whilst it is possible to artificially increase the intracellular ascorbate content of neutrophils isolated from healthy volunteers by incubation with DHA, this does not significantly enhance their functions of chemotaxis and superoxide generation. This suggests that neutrophils isolated from individuals with saturating plasma ascorbate concentrations already contained sufficient intracellular ascorbate to carry out these functions. We did, however, show that co-incubation of neutrophils with ascorbate in the high physiological range (i.e., 200 μmol/L) did attenuate PMA-stimulated NET generation, consistent with another study [22], and may support a role for ascorbate in protecting against inflammatory and autoimmune conditions [19]. Therefore, it is critical to ensure an adequate daily intake of vitamin C to maintain plasma ascorbate concentrations sufficient to support optimal neutrophil function. Although saturation of neutrophils can be achieved by consumption of around 100 mg/day in healthy adults [25,26], we observed decreased NET formation in the presence of 200 µmol/l ascorbate, which is only achieved during peak dietary uptake of gram doses of ascorbate [31].
